# Impact of organizational context on patient outcomes in a proactive primary care program:a longitudinal observational study

**DOI:** 10.1186/s12877-021-02539-6

**Published:** 2021-10-19

**Authors:** Linda C. Smit, Niek J. De Wit, Meggie L. Nieuwenhuizen, Marieke J. Schuurmans, Nienke Bleijenberg

**Affiliations:** 1 Research Centre for Healthy and Sustainable Living, University of Applied Sciences Utrecht, Heidelberglaan 7, Utrecht, 3584 CS The Netherlands; 2 Department of General Practice, Division Julius Center for Health Sciences and Primary Care, University Medical Center Utrecht, Utrecht, 3508 GA The Netherlands; 3 Education Center, UMC Utrecht Academy, University Medical Center Utrecht, Utrecht, 3508 GA The Netherlands; 4 Department of Nursing Science, Julius Center for Health Sciences and Primary Care, University Medical Center Utrecht, Utrecht, 3508 GA The Netherlands

**Keywords:** Primary care, Proactive care program, Implementation, Organizational context, Older people

## Abstract

**Background:**

The effectiveness of health care interventions is co-determined by contextual factors. Unknown is the extent of this impact on patient outcomes. Therefore, the aim of this study is to explore which characteristics of general practices are associated with patient outcomes in a proactive primary care program, the U-PROFIT 2.0.

**Methods:**

A longitudinal observational study was conducted from January 2016 till October 2017. Two questionnaires were send out, one to collect characteristics of general practices such as practice neighbourhood socio-economic status, general practice versus healthcare centre (involving multiple primary care professionals), and professional- frail older patient ratio per practice of general practitioners and practice nurses. Regarding delivering the program, the practice or district nurse who delivered the program, number of years since the start of the implementation, and choice of age threshold for frailty screening were collected. Patient outcomes collected by the second questionnaire and send to frail patients were daily functioning, hospital admissions, emergency department visits, and general practice out-of-hours consultations. Linear and generalized linear mixed models were used.

**Results:**

A total of 827 frail older people were included at baseline. Delivery of the program by a district nurse compared to a practice nurse was significantly associated with a decrease in daily functioning on patient-level (β = 2.19; *P* = < 0.001). Duration since implementation of 3 years compared to 9 years was significantly associated with less out-of-hours consultations to a general practice (OR 0.11; *P* = 0.001). Applying frailty screening from the age of 75 compared to those targeted from the age of 60 showed a significant increase in emergency visits (OR 5.26; *P* = 0.03).

**Conclusion:**

Three associations regarding the organizational context 1) the nurse who delivered the program, 2) the number of years the program was implemented and 3) the age threshold for defining a frail patient are significant and clinically relevant for frail patients that receive a proactive primary care program. In general, contextual factors need more attention when implementing complex primary care programs which can result in better balanced choices to enhance effective proactive care for older people living in the community.

**Supplementary Information:**

The online version contains supplementary material available at 10.1186/s12877-021-02539-6.

## Introduction

Due to the rapid increase of the number of frail older people with complex care needs, health care service utilization and costs increase, urging health care systems to innovate care for older people [1, 2]. Driven by the high costs, national Dutch health policies’ key target is to avoid institutionalization in residential care or nursing homes [3]. Therefore, frail older people with complex care needs in multiple domains are living at home as long as possible [3–6]. As a consequence, home has become the hub of care where district nurses play a pivotal role in providing healthcare as well as the general practitioners, the so called gate keepers [2]. To act upon this, many proactive, integrated care programs for frail older people living in the community have been evaluated and implemented in clinical practice in the last decade. Proactive, integrated care interventions are defined as an organizational process of coordination aiming to achieve seamless and continuous care, tailored to the patient’s needs (based on a holistic view of the patient) with a focus on maintaining independence and prevent of functional decline [7–9]. A proactive integrated care program links the curative medical domain to areas like prevention, mental health, housing and welfare, and therefore requires an interprofessional collaboration [10, 11]. The original U-PROFIT program was designed as an elderly care research project, to evaluate an innovative program for the elderly care in The Netherlands [12, 13]. The program showed a small but significant effect on daily functioning [12]. Furthermore, older people and professionals were satisfied with the program as it provided more personalized, integrated and less fragmented care [14]. Based on the outcome of the evaluation, the program was modified to U-PROFIT 2.0, including social work and district nursing, and regionally implemented as a care delivery program.

Most research regarding these complex interventions assumes a distinction between intervention and context, however many health interventions are intended to modify contexts and thereby become part of the context in which health is produced [15]. Understanding the influence of context is necessary to explain why certain patient outcomes are achieved or simply failed to generalize study findings to different settings [15–17]. In literature, several unique frameworks address contextual factors [3]. Although they do take context into account, no single framework is sufficiently comprehensive about the definition and application of context [17–19]. Context interacts with the place and setting where an intervention is delivered (e.g. primary care setting) according to the context and implementation of complex interventions (CICI) framework [6]. The organizational environment is part of this setting, also referred as organizational support, which includes the organization of work, staff workload and staff training [15, 17, 20, 21].

To understand the impact of the organizational context in interpreting the findings of a complex intervention such as the U-PROFIT 2.0 and generalizing beyond is necessary [15, 22]. Therefore, the aim of this study is to explore which characteristics of general practices are associated with patient outcomes in a proactive primary care program, the U-PROFIT 2.0.

## Methods

### Design and setting

A longitudinal observational study of twelve months was conducted among frail older people in the region of Utrecht in the Netherlands from January 2016 till October 2017. The U-PROFIT 2.0 program was implemented in seven overarching general practices, which consisted of seventeen local general practitioners (GPs) providing proactive primary care to approximately 24,885 patients aged 60 and older. The mean number of GP by practice is therefore quite low (2-3 GPs). With more than 340,000 inhabitants, Utrecht is the fourth biggest city in the Netherlands.

### Proactive primary care program for older people; U-PROFIT 2.0

**Fig. 1:**
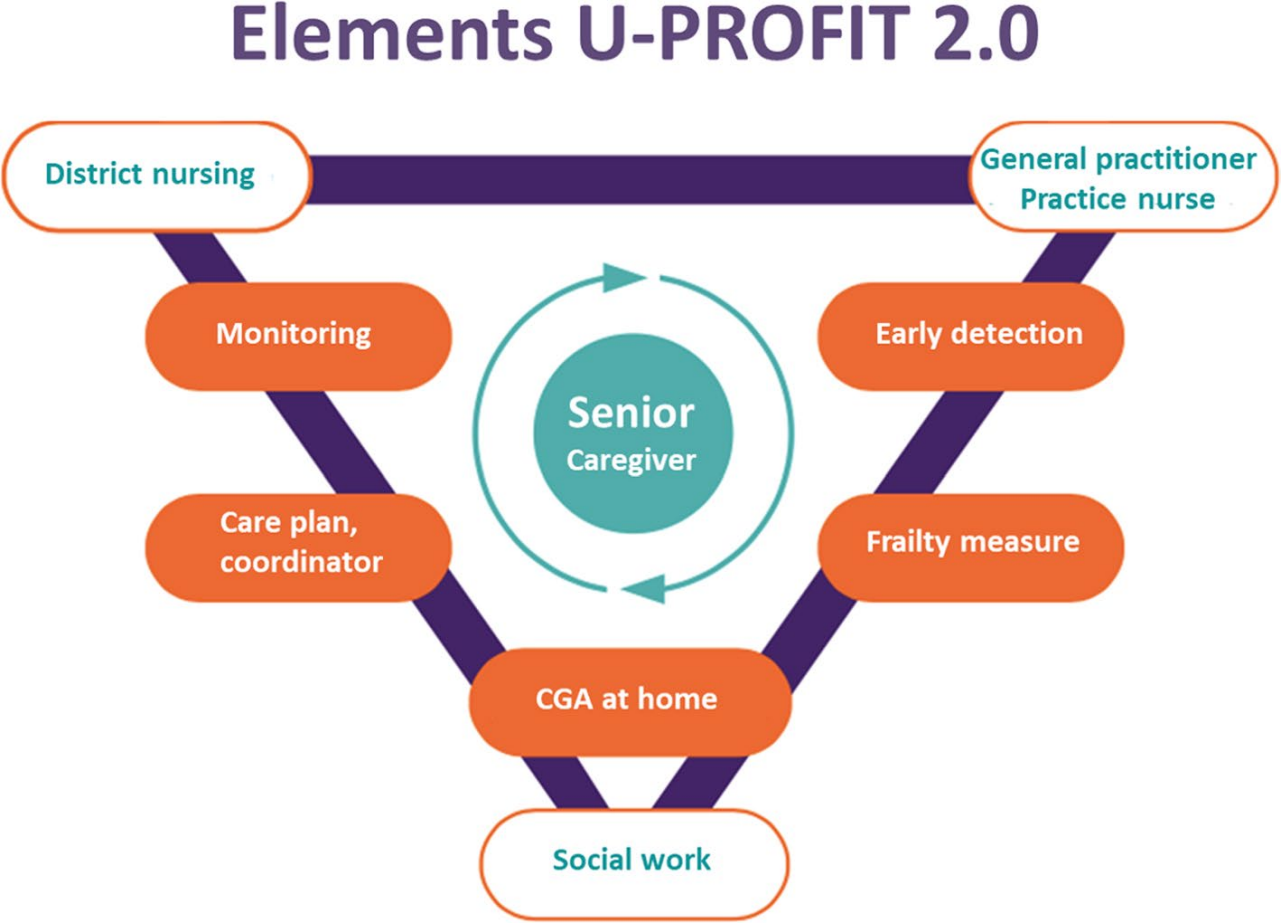
Schematic depiction of U-PROFIT 2.0 program

The proactive primary care program U-PROFIT 2.0 (Fig. 1) is a complex intervention as it consists of multiple elements involving multiple providers [23]. The U-PROFIT 2.0 program was based on the previous developed U-PROFIT 1.0 program (see Table 1) and consists of two parts.

**Table 1:** Description of the U-PROFIT 1.0 and U-PROFIT 2.0 Program

U-PROFIT 1.0 [12, 13]	U-PROFIT 2.0	
1. **Frailty assessment.** • The level of frailty in patients at risk were selected by U-PRIM and further explored with the Groningen Frailty Indicator questionnaire (GFI) which was send by a practice nurse. The U-PRIM software application is an electronic monitoring system aiming at identification of older patients at increased risk of frailty in routine health care data. The software is based on periodic screening for relevant risk factors in the EMRs of the general practice. U-PRIM screens for three core risk factors in patients aged 60 years or older. These are also the eligibility criteria of the U-PROFIT trial as described earlier (multimorbidity, polypharmacy and a care gap). The GFI, a 15-item validated questionnaire that assesses frailty from a functional ADL/ IADL perspective on four domains: physical, cognitive, social and psychological. We chose a score of 4 or higher as the relevant cut-off for the selection of patients that should be visited for a comprehensive geriatric assessment. 2. **Comprehensive Geriatric Assessment at home (CGA)** • A CGA at home is conducted by a registered practice nurse. During this home visit, the practice nurse focuses on patients’ health problems and needs in a structured manner based on the outcome of the frailty assessment 3. **Tailor-made care plan** • In collaboration with the GP, the practice nurse will prepare a tailor-made care plan based on the outcome of step 2. This tailor-made care plan consists of interventions derived from evidence-based care plans developed by the research team, practice nu 4. **Monitoring** • Care coordination and follow-up were provided by the practice nurse.	**1. Frailty assessment.** • The level of frailty in patients at risk were selected by U-PRIM and further explored with the GFI which was send by a practice nurse. 2. **Comprehensive Geriatric Assessment at home (CGA)** • A CGA at home is conducted *by a registered practice nurse or district nurse*. During this home visit, *the practice nurse or district nurse* focuses on patients’ health problems and needs in a structured manner based on the outcome of the frailty assessment 3. **Tailor-made care plan** • *In collaboration with the GP, district nursing and social work, the practice nurse or district nurse*, will prepare a tailor-made care plan based on the outcome of step 2. This tailor-made care plan consists of interventions derived from evidence-based care plans developed by the research team, practice nu 4. **Monitoring** • Care coordination and follow-up were provided by either the *district nurse, practice nurse or social worker* which was based on the needs of the patient.	**U-PRIM U-CARE**

First, the program started with the U-PRIM screening and assessment to identify potentially frail older people [12, 24]. The U-PRIM screening was based on routine care data using automated risk-based detection within electronic medical records [12, 24]. A patient was included when the patient reached the age of 60 or older and met one of the following three criteria: 1) polypharmacy (five or more medications in chronic use); 2) multimorbidity defined as the Frailty Index score of 0.20 or greater which indicates potential frailty (amount of health deficits divided by the maximum of possible health deficits, a score between 0 and 1 represents the number of deficits present divided by the total number of deficits) [25, 26]; 3) consultation gap defined as older people who had not been with the general practitioner (except flu vaccination) for more than 3 years with the aim to screen potentially avoidance of primary care [12]. To identify frail older people, the Groningen Frailty Indicator (GFI) questionnaire was sent to those who were identified by the U-PRIM [27]. Patients were identified as frail when the GFI score was 4 or higher (scale 0-15) [27].

In the second part of the program (U-Care) a Comprehensive Geriatric Assessment (CGA) at home was conducted. Each general practice decided if either specially trained practice nurses or district nurses (see Table 2 for their specific roles in the Dutch primary care delivering) conducted the CGA and further delivering of the U-PROFIT 2.0 program, which was a first modification compared to the U-PROFIT 1.0. Based on the CGA, the interventionist developed a tailor-made care plan in consultation with the patient, and if needed, a social worker and GP. Care coordination and follow-up were provided by either the interventionists or social worker which was based on the needs of the patient. The inclusion of social work was a second modification compared to the U-PROFIT 1.0. Furthermore, regular meetings between these professionals were set up. Compared with the previous U-PROFIT program [12, 13], the U-PROFIT 2.0 program consisted of a more close and integrated care collaboration between the general practice, the district nurse and social care professionals. U-PROFIT 2.0 was implemented within routine primary care without research intentions to measure intervention effectiveness as this was already done based on the U-PROFIT 1.0 [12, 13]. In this light, some adaptations regarding the intervention elements were made by organisations themselves to run the program in the context were it was implemented. For example, not every general practice started with the frailty screening from the age of 60 years but from 75 years.

**Table 2:** Description role and tasks district nurse and practice nurse

**District nurse**	Dutch district nurses are registered nurses with a bachelor’s degree and provide nursing care to the individual patient at home [28]. District nurses are involved in illness prevention, recovery care after an illness or after hospitalization, care for the chronically ill and terminal/palliative care. This also includes care planning and evaluation, caseload management, symptom control and advice, promoting self-management, reassessment of needs, handovers, and administration [6, 29–31]. District nursing is part of home care [32]. Home care also includes care, guidance and domestic help. Home care is provided by home care organizations, some of them also provide long-term care [31]. District nurses are employed by a home care organization and have an important role in care coordination and collaboration with other professionals [31]. They carry out the highly complex care themselves, maintain contacts with other care providers as ultimately responsible and coach other nursing staff on their teams, i.e., certified nursing assistants and aids [33].
**Practice nurse**	Dutch practice nurses are registered nurses with a bachelor’s degree and are employee within a general practice [31, 32]. Practice nurses carry out tasks delegated by the general practitioner such as, taking an anamnesis, physical examination, providing information as well as instruction and education, care planning and evaluation, supporting the general practitioner with oncological -,palliative care as well as care for the chronically ill and older people, drawing up protocols and motivating patients with lifestyle changes [31, 32]. This kind of care by practice nurses will continue to fall under the general practice care and under the supervision of a general practitioner. Practice nurses provide care after the diagnoses has been made by the general practitioner and are involved in the protocolled medical care. The practice nurse holds consultation hours in the general practice and visits the patient to a limited extent at home [31].

### Exposure: characteristics of the general practices

In total, seven characteristics of general practices were measured of which four at general practice level and three regarding the choices made in the local delivery of the U-PROFIT program. We considered the following general practices characteristics; 1) practice neighbourhood socio-economic status (SES) based on the postal areas determined using the Netherlands Institute for Social Research status scores [34], 2) general practice versus healthcare centre in which a healthcare centre included other health or social professionals besides the GP and practice nurse, 3) professional-patient ratio per practice based on the full-time equivalent employment of general practitioners per practice in relation to the total frail patient population they serve and 4) professional-patient ratio per practice based on the full-time equivalent employment of practice nurses per practice in relation to the total frail patient population they serve. Regarding the delivery of the program, the following three factors were collected; 1) the number of years that U-PROFIT 2.0 was implemented, 2) if either the practice nurse or district nurse was in the lead in delivering the U-PROFIT 2.0, 3) and choice of age threshold for frailty screening either 60 years and older or 75 years and older [35]. The data was collected using a questionnaire which was administrated only at baseline to all general practices delivering the U-PROFIT 2.0 and filled in by the practice nurse.

### Outcomes

Four outcomes were determined. First, (instrumental) activities of daily living ((I)ADL) dependency was defined as an increase in depending on someone else when performing (instrumental) activities of daily living. The daily functioning of the older patients was measured by validated questions of The Groningen Activity Restriction Scale (GARS) at baseline and after 12 months follow-up [36]. The GARS comprise of 18 questions about the degree to which someone is able to perform ADL (11 questions) and IADL activities (seven questions) independently. The four response options are: 1) ‘Yes, I can do it fully independently without any difficulty’, 2) ‘Yes, I can do it fully independently but with some difficulty’, 3) ‘Yes, I can do it fully independently but with great difficulty’, 4) ‘No, I cannot do it fully independently, I can only do it with someone’s help’. The results were dichotomized into being independent (options 1-3) or dependent (option 4), as described in the GARS manual [37]. Therefore, the GARS score ranged from 18 to 36, where a higher score indicated more dependency. This way of analyzing was chosen because losing one’s independence is particularly critical and has a higher impact on people’s lives than having difficulties (without dependency) in performing (I)ADL [38].

Second, third and fourth outcome were hospital admission within twelve months, visit to an emergency (ER) department within twelve months, and the general practice out-of-hours consultation within twelve months [12, 39]. Questions measuring these outcomes were formulated as: “In the past twelve months, have you been admitted to the hospital?”; “In the last twelve months, have you been admitted to the emergency department?”; “In the last twelve months, have you needed a visit to- or from- the general practitioner in the evening, night or weekend?”. All three outcomes included two response options: 1) “yes” or 2) “no”. The abovementioned outcomes were collected with a self-reported questionnaire that was administrated by practice nurses at baseline and twelve months after. Patients completed the questionnaires with or without help of a care provider or practice nurse.

### Covariates

Demographic information on age, gender, marital status, country of origin, and education. Polypharmacy and the hours of district nursing care per week were also collected by the self-reported questionnaire. Marital status was categorized into being married or living together, divorced, widowed, and unmarried. Educational level was categorized as low (upper secondary education or less), average (post-secondary non-tertiary education), and high (tertiary or university education) based on the International Classification of Education [40]. Polypharmacy defined as a patient who had five or more medications in chronic use and the hours of district nursing care per week were also collected covariates as they are related to disability [41, 42].

### Statistical analysis

Case mean substitution was applied for the GARS when less than 50% of the total number of items were missing [37, 43]. Non-response analysis was performed on age, gender, marital status, educational status, hours district nursing per week, polypharmacy, and GARS score at baseline to compare respondents and non-respondents at follow up by Chi-square test for binominal outcomes and Mann-Withney U-test for continuous outcomes. Statistical significance was set at *P* ≤ 0.05 (two-tailed). Multicollinearity was assessed by a correlation matrix.

To determine the association between general practices characteristics and the daily functioning linear mixed model (LMM) for a continuous outcome was used. To determine the association between exposure variable on hospital admission, ER visit, and GP out-of hours consultation, generalized linear mixed models (GLMM) for dichotomized outcomes were used. LMM and GLMMs consider all available data points, including patients who missed the follow-up [44]. First, associations of exposure variables with daily functioning, hospital admission, ER visit, and GP out-of hours consultation were examined in a stepwise backward multivariable model, including a random intercept for subjects to account for the repeated measurements, and a fixed intercept for each contextual factor and covariates. Second, the effect over time for daily functioning, hospital admission, ER visit, and GP out-of hours consultation at 12 months follow-up was assessed as a fixed factor in the final models. Third, the interaction between time and baseline covariates such as age, gender, marital status, country of origin, education, district nursing per week and polypharmacy was assessed. All models were adjusted for covariates reported to be related to disability, such as gender, education, socioeconomic status, hours of district nursing per week, and polypharmacy [42]. Model fit was assessed using the − 2 Log-likelihood ratio for GLMM analyses and Akaike’s Information Criterium for LMM analysis. Exposure variables with a significance level of *P* ≤ 0.05 (two-tailed) were considered as statistically significant associated with the outcome. Results were reported as beta coefficients for the LMM and Odds Ratios for GLMM with standard errors and 95% confidence intervals (CI). All analyses were performed using the statistical program IBM SPSS Statistics for Windows (version 24.0; IBM Corp).

## Results

### Study population

**Fig. 2:**
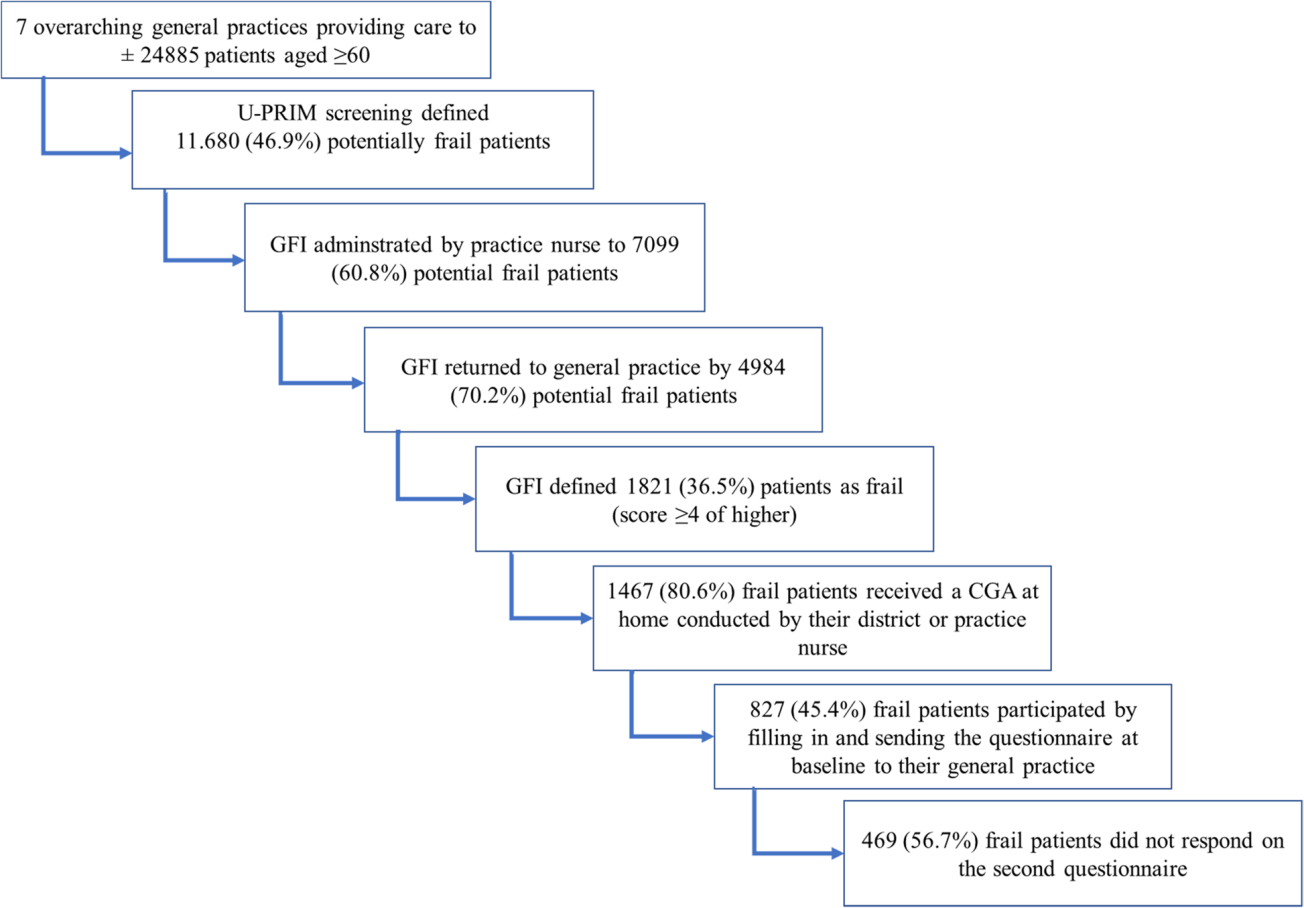
Flowchart of the numbers of patient recruitment and final study population. Notes: The U-PRIM defined potential frail patients. However, not all of these patients lived in the community but in f.e. assisted living facilities. These patients as well as terminal ill patients, although still present in the electronic medical records of the general practice, were not approached as the program focused on community dwelling older people. Furthermore, not all frail patients received a CGA at home. The reason was that frail patients refused to the CGA at home at the time the practice or district nurse called these patients to make an appointment

A total of 827 (45.4%) patients identified as frail by the U-PRIM followed by the GFI (score ≥ 4 of higher) participated and filled in the study questionnaire at baseline (see Fig. 2). Of the included patients, 545 (65.9%) were female, the mean age was 80.0 years (SD 7.3), 42.6% lived alone and polypharmacy was observed in 44.1% (see Table 3, and Additional file 1 for baseline characteristics per practice).

**Table 3:** Baseline practice and patient characteristics

	Total ^**a**^
**Practice**	
Number of older people ≥60 years	24,885
Number of potential frail people aged ≥60 years	11,680
**Participant**	
Gender, *N (%)*	
*Male*	282 (34.1)
*Female*	545 (65.9)
Age, mean ± SD	80.0 ± 7.3
Dutch origin, *N (%)*	747 (90.3)
Marital status, *N (%)*	
*Married*	344 (41.6)
*Widow /widower/partner deceased*	352 (42.6)
*Divorced*	70 (8.5)
*Single*	53 (6.4)
*Sustainable living/unmarried*	7 (0.9)
Education, *N (%)*^b^	
*Low*	116 (14.0)
*Moderate*	530 (64.1)
*High*	172 (20.8)
GARS, median (IQR) (range 18-36)	20 (5.0)
Daily activities problems, *N (%)*	
*None/barely*	485 (58.7)
*Moderate*	186 (22.5)
*Serious*	136 (16.4)
Visits general practitioner, *N (%)*^*c*^	
*0-1 times*	141 (16.9)
*2-3 times*	273 (33.0)
*4-6 times*	228 (27.6)
*> 6 times*	158 (19.1)
Medicines on receipt, *N (%)*	
*0-1 medicines*	72 (8.7)
*2-3medicines*	146 (17.7)
*4-5 medicines*	225 (27.2)
*> 5 medicines*	365 (44.1)
District nursing, *N (%)*^*d*^	
*None*	635 (76.8)
*< 2 h/week*	56 (6.8)
*2-3 h/week*	52 (6.3)
*3-7 h/week*	48 (5.8)
*> 7 h/week*	22 (2.7)
Hospital admission, *N (%)*^c^	208 (25.2)
ER visits, *N (%)*^c^	170 (20.6)
Nursing home admission, *N (%)*^*c*^	48 (5.8)
GP out-of-hours consultation^c,e^	170 (20.6)

*Notes*: *IQR=* interquartile range, *SD=* standard deviation, *FTE=* full-time equivalent

^a^ *N* = 7 general practices, *n* = 827 participants. ^b^ Low = primary school or less, moderate = more than primary school, craft school or secondary school, high = more than secondary school. ^c^ Last twelve months. ^d^ Hours of district nursing per week. **e** General practitioner out of hours consulation during evenings, nights and weekends

At twelve months follow-up, only 469 frail older people participated and filled in the questionnaire, resulting in a 358 (43.3%) drop out (Additional file 2). Reasons for drop-out were relocation to another GP, death refusal to participate feeling too weak to participate, and unknown reasons. A non-response analysis showed that non-responders had a significant higher GARS score at baseline compared to the responders (*P* < .001). Compared to responders, non-responders appeared to have more hours of district nursing per week (*P* < .001).

The median GARS score at baseline and at follow-up for limitations in daily functioning was 20 (IQR 4.0 at baseline and IQR at follow-up 5.0). The percentage of patients with at least one hospital admissions in the previous 12 months was at baseline 25.2% and at follow-up 28.4% respectively. The percentage of patients with at least one ER visit in the previous 12 months was at baseline 20.6 and 20.9% at follow-up. The percentage of patients with at least one a GP out-of-hours consultation in the previous 12 months was at baseline 20.6, and 25.9% at follow-up respectively.

### Outcomes

The results of the multivariable model for daily functioning hospital admission, ER visit, and GP out-of hours consultation are presented in Tables 4, 5 and 6. A significant association was observed between the delivery of the U-PROFIT intervention by a district nurse compared to a practice nurse on a lower daily functioning at patient level (increase of 2.19 points on the GARS score; CI 1.03 to 3.36; *P* = < 0.001). Furthermore, a higher age at screening (75 plus compared to 60 plus) was associated with a significant higher odds ratio on ER visits (OR 5.26; CI 1.17- 23.60; *P* = 0.03). An increase in practice nurse-patient ratio (i.e. number of patients per 1 FTE practice nurse) appeared to be associated with fewer ER visits (OR 0.99; CI 1.00 to 1.00; *p* = 0.01). A reduction in GP out-of-hours consultations was observed when the UPROFIT intervention was implemented 3 years compared to 9 years (OR 0.11; CI-0.03 to 0.39; *P* = 0.001). No contextual factors were significantly associated with hospital admissions. Furthermore, no significant interactions of time with patient characteristics were found that could explain possible inequalities in health over time.

**Table 4:** Univariable and multivariable LMM between general practice characteristics and daily functioning

	Daily functioning							
	Crude estimates				Adjusted estimates			
	***Β***	***SE***	CI (95%)	***P value***	***β***	***SE***	CI (95%)	***P value***
**SES score**	−.62	.14	−.88--.35	<.001				
**Health centre**	1.04	.20	.65-1.44	<.001				
**1 FTE GP on population ratio**	−.002	0.001	−.003- 000	.02				
**1 FTE PN on population ratio**	.000	.000	−.001-000	0.01				
**Om U implemented**								
*3 years* vs. *9 years*	.23	.37	−.41-1.06	.39				
*4 years* vs. *9 years*	−.74	.31	−1.35- -.13	.02				
**Professional who delivered the intervention**								
*District nurse* vs. *practice nurse*	82	.21	.42- 1.22	<.001	2.19	.59	1.03-3.36	<.001
**Age of screening**								
*75 years* vs. *60 years*	−.29	.20	−.69-.10	.15				

*Notes*: *SES=* socio-economic status, *FTE=* full-time equivalent employment. *P* < .05, __ *P* < .01, ___ *P* < .001. Adjusted estimates adjusted for age, sex, education, polypharmacy and hours of district nursing per week. No significant adjusted estimates were found for the following contextual factors; SES score, health centre, FTE GPs population ratio, FTE PNs population ratio, U-PROFIT implemented, age of screening

**Table 5:** Univariable GLMM between general practice characteristics and hospital admissions, ER visits, and GP out-of hours consultations

	Hospital admission				ER visit				GP out-of hours consultation ^**a**^			
	***Β***	SE	CI (95%)	***P value***	***β***	***SE***	CI (95%)	***P value***	***β***	***SE***	CI (95%)	***P value***
**SES score**	.02	.09	.86-1.20	.857	.10	.09	.93-1.32	.269	−.12	.10	.74-1.07	.213
**Health centre**	−.38	.14	.53-.90	.01	−.25	.14	.59-1.02	.074	−.05	.14	.72-1.26	.731
**FTE GPs population ratio**	.002	.000	1.001-1.003	<.001	.001	.000	1.000-1.002	.009	.001	.000	1.000-1.002	.014
**FTE PNs population ratio**	.000	.000	1.000-1.000	<.001	.000	.000	1.000-1.000	.049	.000	.000	1.000-1.000	.158
**Om U implemented**												
*3 years* vs. *9 years*	.51	.27	.99-2.83	.005	1.03	.34	1.43-5.44	.003	1.14	.38	1.50-6.56	.002
*4 years* vs. *9 years*	−.36	.18	.49-1.00	.048	−.23	.20	.54-1.16	.796	−.40	.19	.46-.99	.042
**Professional who delivered the intervention**												
*District nurse* vs. *practice nurse*	−.41	.13	.52-.85	.001	−.29	.14	.57-.98	.033	−.17	.14	.64-1.11	.228
**Age of screening**												
*75 years* vs. *60 years*	.34	.13	1.09-1.83	.006	.13	.14	.87-1.49	.344	. 11	.14	.84-1.47	.452

*Notes*: *SES=* Socio-economic status, *FTE=* Full-time equivalent employment. *ER*= emergency room; *P* < .05, __ *P* < .01, ___ *P* < .001

**Table 6:** Multivariable GLMMs between general practice characteristics and ER visits, and GP out-of hours consultations

	ER visits				GP out-of-hours consultation			
	***β***	***SE***	CI (95%)	***p value***	***β***	***SE***	CI (95%)	***P value***
**FTE PNs population ratio**	−0.001	0.001	−0.002-0.00	.01				
**Om U implemented**								
*3 years* vs. *9 years*					−2.22	0.65	−3.51- 0.94	.001
*4 years* vs. *9 years*					−0.08	0.84	−1.73-1.57	.92
**Age of screening**								
*75 years* vs. *60 years*	1.66	0.76	0.16-3.16	.03				

*Notes*: *SES=* socio-economic status, *FTE=* full-time equivalent employment. *P* < .05, __ *P* < .01, ___ *P* < .001. Adjusted estimates adjusted for age, sex, education, polypharmacy and hours of district nursing per week. No significant associated outcomes for hospital admissions were observed. No significant associations were found for the following contextual factors; SES score, health centre, FTE GPs population ratio, professional who delivered the intervention

## Discussion

This study explored the impact of general practices characteristics on daily functioning, hospital admissions, ER visits and GP out-of-hours consultations in a complex proactive primary care program. We observed that 1) the delivery of the UPROFIT 2.0 program by a district nurse compared to a practice nurse was associated with a higher level of dependency in daily functioning, 2) when the choice was made to screen potential frail older people within the UPROFIT 2.0 program from the age of 75 compared to the choice of screening from the age of 60 years, a significant higher odds on ER visits was observed, 3) if the UPROFIT 2.0 program was implemented 3 years ago compared to 9 years ago, this was significantly associated with fewer GP out-of-hours consultations.

The delivery of the UPROFIT 2.0 program by a district nurse compared to a practice nurse was associated with a higher level of dependency on daily functioning. In clinical practice, there is variation in the type of patient to whom the different nurses deliver care. Practice nurses generally provide care to older people with chronic conditions, while district nurses provide care to older people with complex and multiple care needs with IADL and ADL impact [6, 45]. The association we identified in this study may reflect the normal variation in patient outcomes. Older people receiving care from district nurses are less likely to improve on patient outcomes than older people who only visit their practice nurse. In this light, the choice to deliver the UPROFIT 2.0 program by either a practice nurse or a district nurse should may be not be made on practice level but patient level. However, more research, preferably of qualitative nature, is recommended to unravel how coordination of care planning ends up with the practice or district nurse and what the (dis)advantages are in care delivering.

This study showed that screening potential frail older people from the age of 75 compared to the choice of screening from the age of 60 years showed a significant increase in ER visits. We were not surprised by this finding as with age, the disease burden increases (e.g. biological ageing) [46]. There is no consensus in the literature about the optimal cut-off age for frailty screening [47]. However, in 2013, a consensus meeting of six societies called for screening of all persons 70 years and older for frailty [48]. In our study, the frailty screening was the first step in the proactive care approach, and GP practices determined the age threshold (e.g. either 60+ or 75+) based on their caseload and number of patients with a low SES. Low SES is defined as a risk factor on the rate of biological ageing, which is a fundamental pathway linking SES and health [49]. Furthermore, lowering the age threshold (to 60+), will possibly increase the number of potential frail older people and place a burden of administrative work of the health care professionals, which is a barrier to program implementation [50]. This finding suggests that screening above 70 years with SES moderate-high and at age 60 years for those with a low SES is an alternative choice in delivering proactive care for older people.

Our study showed that when the intervention was implemented less long, this was significantly associated with less GP out-of-hours consultations. This may indicate that the effect of the intervention will fade over time. Similar proactive primary-care programs published in literature paid no or little attention to the training of interventionists which could inhibit successful implementation [16, 51]. The review of Lorthios-Guilledroit et al., (2018) revealed that training can be seen as an opportunity for professionals to become informed about program fidelity, to learn about the program’s target population, and to practice the required skills [52]. Moreover, training can also increase professionals’ confidence in their ability to deliver the program [53]. More research is needed to examine the effect of continuous education on the sustainability of outcomes within complex (primary) healthcare interventions.

Concerning the choices to be made by a general practice (f.e. frailty screening from the age of 75; district nurse or practice nurse providing the program as interventionist, providing training for interventionists on regular basis), the context in which the GP practice is delivering care is important in decision-making processes, which already starts during the development phase of the intervention [16, 54–56]. The UPROFIT program can therefore be conceived as an ‘event in a system’ in order to generate a complete understanding of the relationship between the UPROFIT and its context [15] Furthermore, the complex care needs of older people targeted by an intervention will also differ from one context to another [57], meaning that the same intervention may have different consequences if implemented in a different setting. Other research methods can enrich our view on the influence of context on primary care programs such as realist evaluations that can determine the relation and interactions between context, mechanisms, and outcomes of implemented programs [58]. In addition, policy makers should embrace also findings from other methods than RCTs in their policy regards proactive care for older people in the community as these findings are more transferable to other settings.

### Strengths and limitations

As far as we have known, this is the first study that examines general practices characteristics of the provided health care for their association with daily functioning, hospital admissions, ER visits and GP out-of-hours consultations in a large, well-defined sample of frail community-dwelling older people. This study had some limitations as well. First, this study had an explorative nature and determined no causality. Second, this study could not account for the reach and fidelity/ adaptation of interventions (the degree that they were delivered and taken up as planned in the targeted group [59, 60]. Understanding the black box of intervention delivery is essential and therefore recommended to take into account in future studies to comprehensively explain the found associations on patient outcomes. Third, this study had a selective inclusion due to the use of self-reported questionnaires [61]. Therefore, a relatively independent population of older people was included. This phenomenon was also observed within the original UPROFIT intervention trial effects [12]. Although the mean age was almost 6 years higher (80.0, sd 7.3) compared to the trial participants (74.2 sd 8.4). Still, the included older people in this study had probably little room for improvement in daily functioning. Moreover, the use of self-report ADL and IADL scales have a low sensitivity for detecting small changes, which could have underestimated our effects [62, 63]. Fourth, due to possible change of recall bias when using self-reported questionnaires, hospital admission, ER visit and GP out-of hours consultation were measured on a dichotomized instead of a continuous scale. Also possible confounders such as length of hospitalization and reason of hospitalization were unfortunately not collected. The use of routine care data can overcome the limitation of recall bias in the future. Fifth, the drop-out rate of our study of 43.3% (*N* = 358) was high and specifically observed in those older people who were more dependent in their daily functioning and has twice as much district nursing. This is in line with the studies of Suijker et al., (2014) and van Dalen et al. (2014), who examined differences between respondents and non-respondents at baseline and indicated that non-respondents had more often ADL dependency and received more home visits from their general practitioner [64, 65]. A plausible consequence of the high non-response in our study is an underestimation of the associations. Note, however, that differences between dropouts after baseline and those who did not drop out were small and that eleven out of fourteen variables were assessed.

## Conclusion

The results of this study showed that three associations regarding the organizational context affected patient outcomes among older people that receive a proactive primary care program 1) the nurse who delivered the intervention, 2) the number of years the intervention was implemented and 3) the cut-off for defining a frail patient. Older people that receive a proactive primary care program. In general, the organizational context needs more attention when implementing complex primary care programs and more research is therefore necessary to explain the found associations. Incorporating the ongoing implementation process in primary care programs can result in better balanced choices to enhance effective proactive care for older people living in the community.

## Supplementary Information

**Additional file 1.** Baseline practice and patient characteristics.

**Additional file 2.** Number of participants at baseline and follow up (twelve months after).

## Data Availability

The datasets used and/or analysed during the current study are available from the corresponding author on reasonable request.
