# Supplementary material for: Impact of organizational context on patient outcomes in a proactive primary care program:a longitudinal observational study
Source: BMC Geriatr. 2021 Oct 19;21:578. doi: 10.1186/s12877-021-02539-6 (PMC8527676; doi:10.1186/s12877-021-02539-6)
Supplement: Supplementary file 1 — Additional file 1. Baseline practice and patient characteristics. [file 12877_2021_2539_MOESM1_ESM.docx]

**Additional file 1. Baseline practice and patient characteristics**

|  | **Total ^a^** | **GP 1^b^:** | **GP 2^c^:** | **GP 3^d^:** | **GP 4^e^:** | **GP 5^f^:** | **GP 6^g^:** | **GP 7^h^:** |
| --- | --- | --- | --- | --- | --- | --- | --- | --- |
| **Practice** |  |  |  |  |  |  |  |  |
| Number of older people ≥ 60 years | 24885 | 1871 | 3597 | 4319 | 4189 | 2974 | 2343 | 5592 |
| Number of potential frail people aged ≥ 60 years | 11680 | 936 | 870 | 2619 | 2813 | 513 | 490 | 3439 |
| Socioeconomic status score^i^ | N.A. | -0.93 | -1.54 | 0.69 | 1.87 | -0.62 | -0.87 | -1.09 |
| Health centre, yes/no | N.A. | Yes | Yes | Yes | Yes | Yes | Yes | No |
| FTE GPs | N.A. | 2.31 | 4.3 | 7.3 | 2.6 | 7.2 | 4.6 | 2.0 |
| 1 FTE GP on population ratio | N.A. | 424.20 | 175.06 | 350.40 | 275.86 | 64.44 | 101.08 | 470.56 |
| FTE PNs | N.A. | 0.84 | 0.56 | 1.63 | 0.40 | 0.70 | 0.80 | 0.50 |
| 1 FTE PN on population ratio | N.A. | 1166.67 | 371.78 | 1569.33 | 903.23 | 662.86 | 581.25 | 2570.00 |
| Years U-PROFIT implemented | N.A. | 3.0 | 4.0 | 9.0 | 9.0 | 9.0 | 9.0 | 9.0 |
| Delivering intervention, PN/PN+DN^j^ | N.A. | PN | PN+DN | PN+DN | PN | PN+DN | PN+DN | PN |
| Age of screening, > 60/ >75 years | N.A. | >60 | >60 | >75 | >60 | >60 | >60 | >75> |
| **Participant** |  |  |  |  |  |  |  |  |
| Gender , *N (%)*  *Male*  *Female* | 282 (34.1)  545 (65.9) | 27 (38.0)  44 (62.0) | 38 (38.1)  60 (61.9) | 31 (32.3)  65 (67.7) | 49 (28.8)  116 (71.2) | 26 (30.2)  60 (69.8) | 22 (32.3)  44 (67.7) | 89 (36.3)  156 (63.7) |
| Age, mean ± SD | 80.0 ± 7.3 | 85.0 ± 7.2 | 73.5 ± 8.1 | 84.2 ± 6.5 | 80.2 ± 7.1 | 76.4 ± 10.0 | 77.9 ± 6.9 | 83.1 ± 5.2 |
| Dutch origin, *N (%)* | 747 (90.3) | 68 (95.8) | 85 (88.5) | 92 (95.8) | 143 (86.7) | 77 (89.5) | 55 (84.6) | 227 (92.7) |
| Marital status, *N (%)*  *Married*  *Widow /widower/partner deceased*  *Divorced*  *Single*  *Sustainable living/unmarried* | 344 (41.6)  352 (42.6)  70 (8.5)  53 (6.4)  7 (0.9) | 20  39 (54.9)  6 (8.5)  6 (8.5)  0 (0.0) | 50  30 (30.6)  12 (12.2)  5 (5.1)  1 (1.0) | 33  52 (54.2)  5 (5.2)  6 (6.25)  0 (0.0) | 52  79 (48.2)  16 (9.8)  17 (10.3)  0 (0.0) | 41  30 (34.9)  11 (12.8)  4 (4.7)  0 (0.0) | 39  15 (22.7)  7 (10.6)  3 (4.6)  2 (3.0) | 109  107 (43.7)  13 (5.3)  12 (4.9)  4 (1.6) |
| Education, *N (%)*^k^  *Low*  *Moderate*  *High* | 116 (14.0)  530 (64.1)  172 (20.8) | 4 (5.8)  47 (68.1)  18 (26.1) | 19 (20.0)  64 (67.4)  12 (12.6) | 10 (10.4)  75 (78.1)  11 (11.5) | 49 (29.9)  102 (62.2)  13 (7.9) | 12 (14.1)  67 (78.8)  6 (7.1) | 11 (16.9)  39 (60.0)  15 (23.1) | 11 (4.5)  136 (55.7)  97 (39.8) |
| GARS, median ( IQR) (range 18-36) | 20 (5.0) | 21 (4.0) | 20 (4.0) | 21 (6.0) | 20 (5.3) | 21 (4.0) | 20 (4.25) | 20 (4.0) |
| Daily activities problems, *N (%)* *None/barely*  *Moderate*  *Serious* | 485 (58.7)  186 (22.5)  136 (16.4) | 39 (55.7)  21 (30.0)  10 (14.3) | 53 (58.9)  20 (22.2)  17 (18.9) | 56 (58.9)  18 (18.9)  21 (22.1) | 91 (56.9)  42 (26.3)  27 (16.9) | 50 (58.8)  18 (21.2)  17 (20.0) | 34 (54.0)  14 (22.2)  15 (23.8) | 162 (66.4)  53 (21.7)  29 (11.9) |
| Visits general practitioner, *N (%)^l^*  *0-1 times*  *2-3 times*  *4-6 times*  *>6 times* | 141 (16.9)  273 (33.0)  228 (27.6)  158 (19.1) | 17 (24.3)  27 (38.6)  18 (25.7)  8 (11.4) | 11 (11.7)  29 (30.9)  24 (25.5)  30 (31.9) | 19 (20.2)  25 (26.6)  26 (27.7)  24 (25.5) | 33 (21.2)  56 (35.9)  35 (22.4)  32 (20.5) | 9 (10.8)  32 (38.6)  18 (21.7)  24 (28.9) | 9 (13.8)  13 (20.0)  25 (38.5)  18 (27.7) | 43 (18.1)  91 (38.2)  82 (34.5)  22 (9.2) |
| Medicines on receipt, *N (%)*   - 1. *medicines*   2. *medicines*   3. *medicines*   *> 5 medicines* | 72 (8.7)  146 (17.7)  225 (27.2)  365 (44.1) | 8 (11.8)  16 (23.5)  29 (42.6)  15 (22.1) | 1 (1.0)  5 (5.2)  15 (15.5)  76 (78.4) | 13 (14.0)  22 (23.7)  24 (25.8)  34 (36.6) | 15 (9.4)  24 (15.1)  41 (25.8)  79 (49.7) | 2 (2.4)  8 (9.5)  22 (26.2)  52 (61.9) | 3 (4.7)  11 (17.2)  15 (23.4)  35 (54.7) | 30 (12.3)  60 (24.7)  79 (32.5)  74 (30.5) |
| District nursing, *N (%)^m^*  *None*  *< 2 hours/week*  *2-3 hours/week*  *3-7 hours/week*  *> 7 hours/week* | 635 (76.8)  56 (6.8)  52 (6.3)  48 (5.8)  22 (2.7) | 37 (52.9)  10 (14.3)  12 (17.1)  9 (12.9)  2 (2.9) | 88 (91.7)  2 (2.1)  3 (3.1)  0 (0.0)  3 (3.1) | 62 (66.0)  9 (9.6)  11 (11.7)  8 (8.5)  4 (4.3) | 122 (77.2)  8 (5.1)  8 (5.1)  16 (10.1)  4 (2.5) | 70 (82.4)  6 (7.1)  4 (4.7)  5 (5.9)  0 (0.0) | 52 (78.8)  6 (9.1)  3 (4.5)  3 (4.5)  2 (3.0) | 204 (83.6)  15 (6.1)  11 (4.5)  7 (2.9)  7 (2.9) |
| Hospital admission, *N (%)*)^l^ | 208 (25.2) | 13 (18.8) | 32 (33.0) | 21 (22.6) | 40 (24.8) | 29 (34.1) | 21 (31.8) | 52 (21.4) |
| ER admission, *N (%)*^l^ | 170 (20.6) | 7 (10.1) | 27 (27.8) | 18 (19.1) | 35 (22.0) | 14 (16.5) | 20 (30.3) | 49 (20.2) |
| Nursing home admission, *N (%)*^l^ | 48 (5.8) | 3 (4.3) | 2 (2.1) | 9 (9.6) | 8 (5.0) | 3 (4.7) | 5 (7.6) | 18 (7.4) |
| GP out-of-hours consultation^l,n^ | 170 (20.6) | 7 (10.1) | 23 (23.7) | 23 (24.7) | 41 (25.5) | 20 (23.5) | 14 (21.5) | 42 (17.3) |

^IQR = interquartile range; SD = standard deviation., FTE= full-time equivalent, PN= practice nurse, DN= district nurse^

^a N= 7 general practices, n = 827 participants. b N= 1 general practice, n= 71 participants. c N= 4 general practices, n= 98 participants. d N= 1 general practice, n= 96 participants. e N= 6 general practices, n= 165 participants. f N= 1 general practice, n= 86 participants. g N= 1 general practice, n= 66 participants. h N= 4 general practices, n= 245 participants^

**^i^** ^Based on ZIP code, Socioeconomic status low = score > 1; medium= score -1 – 1; high = score <-1.^

**^j^** ^Intervention delivered by Practice Nurse or delivered by both Practice Nurse and District Nurse^

**^k^** ^Low = primary school or less, moderate = more than primary school, craft school or secondary school, high = more than secondary school.^

^l Last twelve months.^

^m Hours of district nursing per week.^

^l Special visits general practitioner out of office hours (during evenings, nights and weekends).^
