# Supplementary material for: Impact of organizational context on patient outcomes in a proactive primary care program:a longitudinal observational study
Source: BMC Geriatr. 2021 Oct 19;21:578. doi: 10.1186/s12877-021-02539-6 (PMC8527676; doi:10.1186/s12877-021-02539-6)
Supplement: Supplementary file 2 — Additional file 2. Number of participants at baseline and follow up (twelve months after). [file 12877_2021_2539_MOESM2_ESM.docx]

| General Practice | Number of participants at baseline (%) | Number of participants at follow up (%)^a^ | Number of dropouts (%)^b^ |
| --- | --- | --- | --- |
| 1 | 71 (12.5) | 33 (7.0) | 38 (10.6) |
| 2 | 98 (17.3) | 60 (12.8) | 38 (10.6) |
| 3 | 96 (11.6) | 42 (9.0) | 54 (15.1) |
| 4 | 165 (20.0) | 84 (17.9) | 81 (22.6) |
| 5 | 86 (15.2) | 55 (11.7) | 31 (8.7) |
| 6 | 66 (11.7) | 48 (10.2) | 18 (5.0) |
| 7 | 245 (43.3) | 147 (31.3) | 98 (27.4) |
| Total | 827 (100.0) | 469 (100.0) | 358 (100.0) |

**Additional file 2. Number of participants at baseline and follow up (twelve months after)**

**Notes:** ^a^ Number of participants measured based on all four outcomes (e.g. daily function and care consumption). ^b^ Dropouts reasons: relocation (N= 7), admission to nursing home (N= 3), refusal to participate (N= 7), felt too weak to participate (N=7), deceased (N= 11) and unknown (N= 323).
